# Ureteric stents on extraction strings: a systematic review of literature

**DOI:** 10.1007/s00240-016-0898-1

**Published:** 2016-06-20

**Authors:** Rachel Oliver, Hannah Wells, Olivier Traxer, Thomas Knoll, Omar Aboumarzouk, Chandra S. Biyani, Bhaskar K. Somani, YAU Group

**Affiliations:** 1 Department of Urology, University Hospital of Southampton NHS Foundation Trust, Southampton, SO16 6YD UK; 2 Department of Urology, Tenon Hospital, University Pierre and Marie Curie, Paris, France; 3 Department of Urology, Klinikum Sindelfingen-Böblingen, Sindelfingen, Germany; 4 Urology Fellow, Bristol Urological Institute, Bristol, UK; 5 EAU Young Academic Urologists Group, Arnhem, The Netherlands; 6 Leeds Teaching Hospital NHS Trust, Leeds, UK

**Keywords:** Stent, String, Ureteric, Extraction, Ureteroscopy

## Abstract

Short-term ureteric stents are commonly placed after ureteroscopy. The removal usually entails having a cystoscopy, but recently, endourologists have been using stents with extraction strings attached to them for ease of removal. We wanted to conduct a systematic review of literature looking at the outcomes of ureteric stents with extraction strings attached to them. Our objective was to investigate the use, morbidity, tolerability, complications, associated cost, and patient preference of stents with extraction strings attached to them. All studies in English language (between 1990 and 2015) where stents on extraction strings were either self-removed by patients or removed by physician were included. A total of eight studies (1279 patients) were included, of which 483 (38 %) patients had extraction strings for removal. There seemed to be no overall difference in pain scores or urinary symptoms between patients with and without extraction strings, but nearly 10 % of patients suffered stent dislodgement in the group with extraction strings attached. Overall stent dwell time was lower in patients who had their stents removed via extraction strings, and majority of them were able to remove their stents at home. Our study suggests that stents with extraction strings are easy for patient self-removal and can reduce the stent dwell time for patients, thus reducing the duration of morbidity and physical and financial burden to patients. However, this must be balanced against a risk of stent dislodgement and, hence, may not be a good option in all patients.

## Introduction

Placement of an indwelling ureteral stent following uncomplicated ureteroscopy (URS) for stone disease is currently commonplace, with over three-quarters of urologists reporting this practice [1–4]. Prophylactic stent placement may reduce the risk of ureteric obstruction, symptoms such as clot/fragment colic, and stricture formation following ureteric inflammation from ureteroscopic stone retrieval [5]. However, recent studies have revealed no significant difference in complication or stone-free rates (SFR) between patients with or without post-operative ureteric stents following uncomplicated ureteroscopic stone removal. Furthermore, those with stents were also found to have higher rates of stent-related discomfort or pain and associated lower urinary tract symptoms (LUTS), occurring in up to 90 % of patients with stents [6].

Stent placement and subsequent removal also resulted in higher procedural costs than when a stent was not used [7–11]. In uncomplicated URS, there is no major advantage in utilising routine stents in this group of patients, and there appears to be a high level of morbidity associated with their use. Despite this, the majority of urologists place a stent after uncomplicated URS possibly because of a decrease in the return rate to the emergency department in patients who are stented post-ureteroscopy [12]. This may reflect a lack of clear guidelines over what constitutes uncomplicated URS, and the experiences and preferences of individual urologists [13].

Once ureteral stents are placed, they may be removed via flexible cystoscopy in an outpatient setting (office-based cystoscopy) or cystoscopy in the operating theatre. However, if stents with extraction strings are used, the patients can themselves remove it or a clinician in an outpatient clinic can remove it. A number of modern stents incorporate extraction strings made of fine suture material secured to the distal end of the stent which, when placed, runs through the urethra and is visible at the urethral meatus. The string can then either be left free or be secured to the patient, typically to the mons pubis or thigh in women or to the penis in men [14].

Although extraction strings avoid the need for repeat cystoscopy for stent removal, more than two-thirds of urologists remove stent extraction strings prior to their insertion [2, 14]. The rationale behind this is thought to be due to concerns over perceived risks, including increased LUTS from string irritation, stent dislodgement, infection, stent retention due to patients forgetting to remove stents, broken strings, and lack of strong evidence relating to its safety and tolerability [5, 12–20].

Due to paucity in the available literature, we wanted to conduct a systematic review of literature looking at the outcomes of ureteric stents with extraction strings attached to them.

## Methods

The objective of this systematic review was to investigate the use, morbidity, tolerability, complications, associated cost, and patient preference of stents with extraction strings attached to them.

### Search strategy

The search involved finding relevant studies from MEDLINE, EMBASE, Ovid, the Cochrane Central Register of Controlled Trials, CINAHL, Google Scholar, and individual urological journals between January 1990 and September 2015. Two reviewers (RO and HW) independently identified all studies that fitted the inclusion criteria for this review, and any discrepancy was resolved after adjudication and consensus with the senior author (BKS). The search terms included the following: ‘ureteroscopy’, ‘flexible ureteroscopy’, ‘renal’, ‘calculi’, ‘stone(s)’, ‘urolithiasis’, ‘laser’, ‘stents’, ‘thread’, and ‘extraction string’. These terms were combined using Boolean operators (AND, OR) to refine the search.

### Inclusion criteria

The inclusion criteria were for studies written in the English language, reporting on the use of stents with extraction strings, where they were either self-removed or removed by the physician in an outpatient setting. Papers dealing with individual case reports and paediatric patients were not included in our review. If more than one paper were available from the same authors/group including the same patient cohort, and the most updated version was included.

### Data extraction

The studies fitting the inclusion criteria were analysed for the following: journal of publication, period of review, type of study, country of origin, population demographics (age and sex), adverse events, stent dislodgements, associated pain, and urinary symptoms. The data included were filtered into raw numbers, either directly or as a conversion from a percentage of the original study group.

## Results

**Fig. 1:**
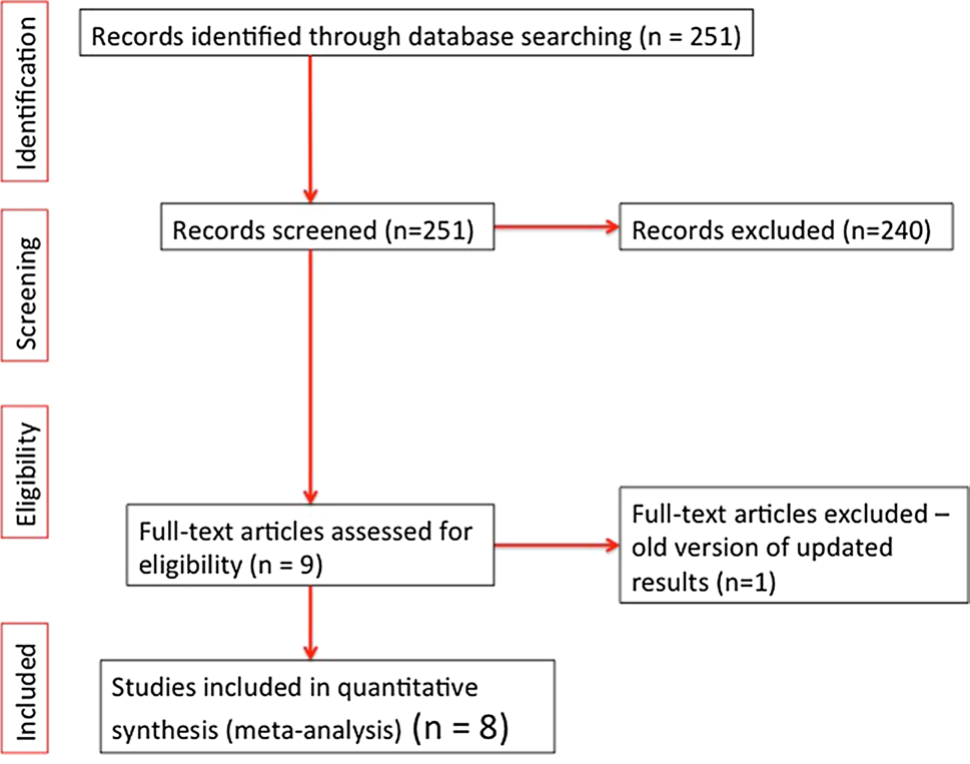
PRISMA flow diagram summarising literature review

A total of 11 studies were identified from the literature search, of which eight fitted the inclusion criteria (Fig. 1). Of these, four articles that were excluded involved one case report [19], one comment [20], one study that looked at extraction string use in children [21], and one paper where an updated article was available from the same authors [14].

### Patient demographics and complications

Eight studies involved 1279 patients, of which 483 (38 %) were patients who had extraction strings for removal, and nearly two-thirds of them had stents, where the extraction string was removed prior to their placement. The mean age was 49 years with a male:female ratio of 9:10 (Table 1). Other baseline characteristics of the eight articles reviewed can be seen in Table 1. All stents were inserted under direct vision plus fluoroscopic guidance or with fluoroscopic guidance alone. Across these articles, the size of these indwelling stents was between 4.7 and 7F.

**Table 1:** Summary of articles assessing use of ureteric stents on extraction strings [5, 12–17, 21]

Author	Study type	Journal	Year	Period of review	Country	No. of patients	M:F	Mean age	Comparator	Strings:no strings
Pryor	Prospective cohort	*J Urol*	1991	–	USA	73	45:28	–	Pain score at removal, urinary symptoms	44:29
								(23–72)		
Bockholt	Retrospective cohort	*BJU Int*	2012	June 2009 –June 2010	USA	181	86:95	45.5	Procedure related events (PREs)	43:138
York	Prospective questionnaire	*J Urol*	2013	May –Dec 2011	New Zealand	50	39:11	51 (21–77)	Pain score, anxiety, complication rate	50:0
Kuehaus	Prospective cohort	*J Urol*	2013	Nov 2011–May 2012	Austria and Germany	124	86:38	47 (20–79)	Pain score at removal	13^a^:111
Barnes	Prospective RCT	*BJU Int*	2014	Oct 2011–May 2013	USA	68^a^	26:42	49	USSQ, pain score, adverse events	33:38
Althaus	Retrospective notes review	*J Urol*	2015	–	USA	512	57:41	54	Dislodgement rates	98:414
Loh-Doyle	Prospective Web-based questionnaire	*J Endourol*	2015	May 2013–Dec 2013	USA	571	183:388	–	Pain score, removal preferences	223:348
Kim	Prospective RCT	*BMC Urology*	2015	July 2012–Nov 2014	Korea	114	78:36	50	USSQ, VAS, patient preference	58:56
Total						1279	600:679			483:796

*RCT* randomised controlled trial, *PPRE* post-procedure related events, *USSQ* ureteric stent symptom questionnaire, *VAS* visual analogue scale

^a^Underpowered

Of the included studies, two were randomized, four were prospective, and the remaining two were retrospective in nature. The two randomised controlled trials compared the use of stents with and without extraction strings attached to them.

The total number of patients that had ureteric stents with extraction strings attached was 483 (range 13–223/study). The mean age was similar across the groups (only adult patients were included in the review analysis) with an overall mean age of 49 years consistent with patients commonly undergoing stone treatment. The male:female ratio shows a slight female preponderance.

The complications for patients with ureteric stents with extraction strings are shown in Table 2. Where available, the results for the comparable groups with ureteric stents without extraction strings are also recorded. The only numeric data for outcomes are in the form of total number of dislodgements and total number of adverse events. ‘Events’ were defined as emergency department visits, unscheduled clinic visits, and telephone calls. There are broadly similar rates of adverse events between the ‘strings’ and ‘no strings’ groups. The articles also looked at pain scores with the stent in situ and upon removal, and the associated urinary symptoms. There seemed to be no overall difference in pain scores or urinary symptoms between patients with and without extraction strings [15].

**Table 2:** Summary of complications across articles in literature review

	Overall number of events		Stent dislodgements		Pain score	Urinary symptoms
	Strings	No strings	Strings	No strings		
Pryor	–	–	–	–	No difference	No difference
Bockholt	16	46	2/43	–		
York	–	–	–	–	Low (strings)	
Kuehaus	–	–	–	–	No difference	
Barnes	13	14	5/33	–	No difference	No difference
Althaus	13	0	13/98	0		
Loh-Doyle	–	–	–	–	No difference	
Kim	–	0	3/58	0		
Total	32 (7.5 %)	60 (8 %)	20/232 (9.9 %)	0		

### Method of stent removal

Majority of patients who had stents with extraction strings were able to remove their stents at home (Table 3) [5, 13]. One patient each in two of these studies did not remove their stent at home due to anxiety [5, 13]. Slightly lower rates of self-stent removal were reported by York et al., with 70 % (*n* = 35) finding removal easy, 24 % (*n* = 12) asking a healthcare professional to remove their stent and 6 % (*n* = 3) asking their spouse to do so [16].

**Table 3:** Summary of outcomes related to tolerability across articles in literature review

	Dwell time (days)		Pain score on removal (0–10)		Patients able to remove own stent at home	Patients who would have strings again
	Strings	No strings	Strings	No strings	Strings only	
Pryor	–	–	3.9	5	–	–
Bockholt	–	–	–	–	42 (97.7 %)	–
York	–	–	2	–	35 (70 %)	38 (75 %)
Kuehaus	–	–	–	–	–	–
Barnes	6.3	10.6	2.5	3.1	32 (97 %)	–
Althaus	–	–	–	–	–	–
Loh-Doyle	–	–	3.7	5.14	–	197 (88.2 %)
Kim	6	6.2	2.9	4.2	–	16/16 (100 %)
Average	6.3	10.6	3.0	4.41	88.2 %	81.6 %

### Stent dislodgement

None of the patients suffered premature stent dislodgement when there was no extraction strings attached, but nearly 10 % of patients suffered stent dislodgement in the group with strings attached (Table 2). This unanticipated early stent removal did not seem to adversely affect the overall outcome or patient safety in these studies. Conversely, Barnes et al. reported one case of stent retention in a patient with a stent without strings who was lost to follow up. This was identified 6-month post-insertion and was removed by office cystoscopy [13].

### Stent dwell time

Overall stent dwell time was lower in patients who had their stents removed via extraction strings. Barnes et al. reported a mean dwell time of 6.3 versus 10.6 days in the strings versus no strings cohorts, respectively (*p* < 0.001) [13].

### Patient preference of stent removal

York et al. reported 75 % of patients would happily remove their own stent again using extraction strings if the need arose in the future (Table 3) [16]. Similarly, a Californian group found that when patient self-removed their stents, 60 % would choose this option again [15]. They also found that the average pain with self-removal was significantly less (*p* < 0.0001) that when removed via formal cystoscopy or by the doctor pulling the string for them. Similarly, when asked which was most important to them regarding stent placement and removal (scale 1–5), patients rated that ‘being informed they are having a stent (4.7)’ most important, followed by ‘method of stent removal (4.6)’, ‘option of general anaesthesia (3.1)’ and ‘being shown a video or diagram of stent removal (2.9)’ [15].

### Cost

Potential cost saving was reported by Barnes et al. when utilising stent extraction strings. Although a formal cost analysis was not performed, it was reported that if all patients in the study removed their own stents using extraction strings, it would result in ~$97,000 saved in cystoscopic removal costs ($243/patient). Also, based on an average 177 mile round trip by patients for cystoscopic stent removal and the cost of driving at $0.40–0.90/mile (based on American Automobile Association estimates), savings in out-of-pocket expense of $68–185/patient were estimated if patients were to remove their own stents at home [13].

## Discussion

### Findings of our study

Ureteric stents have been used to facilitate urinary drainage to bladder since the beginning of the 1960s [17–23]. Although benefits in certain patients are clear, indwelling stents present their own set of problems to the patients while in situ and subsequently during their removal. The standard stent removal for indwelling stents usually requires an elective appointment slot, nursing and medical staff provision, and potentially even a general anaesthesia in some cases. There is also a need for equipment, including a cystoscope, fluid irrigation, camera stack, and stent graspers. Cystoscopy itself is associated with a small risk of morbidity [20]. For patients, travelling to and from the hospital for multiple appointments can be cumbersome and costly [5, 13].

Just having the stent in situ causes discomfort and anxiety resulting in reduced ability to work and loss of earnings [15, 24]. Due to the impact on patients and acute urological services of the adverse effects of indwelling ureteric stents, there has been much research into assessment and reduction of associated morbidity. Validated quality of life measure for stent discomfort is now available, such that studies could investigate factors affecting stent related symptoms and help to develop new technologies to reduce these events [24].

Since the original use of silicone ‘splints’, it has, since, been noted that stents are prone to encrustation and migration. There have been a number of efforts in fields of engineering (stent materials, size, and shape) and pharmacology (alpha blockers) to reduce morbidity associated with indwelling ureteric stents [24]. As none of this research has developed the ‘perfect’ stent or an ideal method of treating the side effects, it seems that reducing the dwell time of stents may be one way forward.

Although majority of urologists place a stent following URS post-stone treatment (up to 80 %), less than a quarter (19–23 %) utilises stent extraction strings in order for patients to remove their own stents at home [5, 14, 15]. This may be due to concerns over the perceived risks of stent extraction strings, such as urinary symptoms from string irritation, infection, stent dislodgement and retention, and the lack of evidence over risks of usage [5, 13, 14]. Surgeon preferences and personal experiences with extraction strings may have an influence on usage, with some urologists using extraction strings far more frequent than others. Bockholt et al. confirmed this in a retrospective study, where majority of stents with extraction strings were used by one out of seven urologists [5]. Total procedure duration was reported to be an average 21.8 min shorter when stent extraction strings were used compared with procedures when they were not used. This possibly reflects the fact that for more challenging cases with greater risk of complications, the surgeon’s not wishing to risk stent dislodgement associated with the use of extraction strings, preferred to use the standard stents without strings [5].

Loh-Doyle and colleagues found that urologists in certain countries more commonly placed stents with strings than in others. The use of extraction strings was reported to be most common in Canada (25.6 %), followed by the United States (12.6 %). None of the 31 respondents from the United Kingdom had used extraction strings [15]. These geographical variations may reflect the overall working culture and possibly surgeon and/or patient attitudes/preferences to use of stent extraction strings in these countries. Although the reasons could be multifactorial, it is also influenced by urological service provision in these countries—for instance, patients in the United States and Canada will often have a much greater distance to travel to visit their urologist than those in the UK.

Studies also revealed that patients often had strong preferences with regard to stent removal method. In one randomized control trial, 202 potential candidates refused to participate, as they did not want to remove their stents themselves (50 % of those approached) [13]. Also, 66 (16.5 %) refused involvement in the study, because they wanted to remove their stents at home. York et al. found patients were anxious about removing their own stent, with a median anxiety score of 5/10. Reasons stated for these were ‘fear or the unknown’, possibility of pain, and fear of the stent getting stuck [16].

In their randomized prospective study, Barnes et al. reported no difference in validated quality of life (QoL) measures between the patients with and without extraction strings, including ‘urinary symptoms’, ‘pain’, ‘general health’ or ‘work performance’ at 1-day post-operatively, 6 days post-operatively, and 6 weeks post-stent removal [13]. This suggests that clinicians concerns over increased urinary symptoms with extraction strings are speculative.

### Advantages of stent on extraction strings

Reported pain outcomes varied between the Barnes et al. and Loh-Doyle et al. studies [13, 15]. With regard to pain upon removal, Barnes et al. found no difference in mean pain scores between groups with and without strings [13]. In contrast, Loh-Doyle et al. reported variation in mean pain scores, with patients who used strings to remove their own stents reporting the lowest mean pain scores [15]. This was similar to mean pain scores for cystoscopic removal in the operating room (OR), suggesting that the use of extraction strings was well-tolerated at the time of stent removal. The use of intra-urethral lidocaine jelly during cystoscopic stent removal may have affected pain scores reported at removal, possibly falsely reducing pain scores compared with self-removal whether no lidocaine jelly was used [14].

Stent dwell time was reported to be significantly lower in patients removing their own stents via extraction strings (Table 3). This was reported to be due to scheduling restraints in arranging appointments for stent removal. The use of extraction strings is advantageous with regard to stent dwell time as patients are able to remove them at home on the date required, with greater convenience. This also gives the patient more control over their removal, which may be preferred by some patients. Also, it is well-reported that indwelling stents negatively impact quality of life and cause troublesome symptoms [25, 26]. Reduced stent dwell time reduces the duration of morbidity and positively impacts patient QoL [26]. As a result of this, it is possible the increased stent dwell time in patients who did not have extraction strings misleadingly increased stent related symptoms and morbidity when compared with those who had extraction strings.

### Disadvantages of stent on extraction strings

Delayed pain appeared to occur most frequently in patients who removed their own stents using strings, although these results did not reach statistical significance. Reasons for this potential increase in delayed pain in patients removing their own stents using extraction strings were unclear. It has been suggested that strings may cause physiological changes, such as trigonal oedema, which leads to delayed pain post-stent removal; however, there are no studies confirming this [15].

The main complication associated with the use of stent extraction strings was reported to be stent dislodgement (Table 2). There were no reported cases of stent dislodgement occurring in patients with stents without extraction strings. The risk of stent dislodgement was four times greater in women than in men [14], presumably due to female hygiene practices and urethral anatomy. Both Barnes and Althaus reported similar rates of stent dislodgement (15 and 13.3 %, respectively), but Bockholt et al. reported significantly lower rates (4.7 %) [5, 13, 14].

Some cases of stent dislodgement occurred in the recovery room [14], highlighting the need for careful patient transfer and monitoring in the immediate post-operative period. Althaus et al. reported two patients removing their stents prematurely without consulting a doctor, emphasising the need for pre-operative patient education regarding the reason for stent placement and aftercare instructions including contacting their urologist if premature stent removal is contemplated. Hence, the decision on the type of stent used or its removal should be based on appropriate patient preference and counselling.

Securing stent extraction strings to the patient did not appear to affect dislodgement rates, although this was not subject to statistical analysis. Neither Bockholt et al. nor Barnes et al. secured extraction strings to the patient externally [5, 13]. Althaus et al. described securing the extraction strings to the penis in men and mons pubis or thigh in women [14]. Despite this variation in technique, Barnes et al. and Althaus et al. reported similar rates of stent dislodgement.

Although the rate of premature inadvertent stent removal in the combined group was relatively low, the use of extraction strings are not advisable in patients whom early stent removal would risk major morbidity. Such cases include those with ureteric perforation, solitary kidney, pyelonephritis, or extrinsic ureteric compression [12, 14, 20].

One episode of stent retention was reported by the American group [13]. The patient failed to attend multiple follow-up appointments, which was identified at 6-month post-stent insertion. Although Barnes et al. report the stent was removed and no adverse outcomes resulted, this emphasises the need for robust follow-up procedures. It could be argued that stents without extraction strings may be forgotten, because, in the absence of pain/urinary symptoms, there is no external reminder of the stent placement. Theoretically, using extraction strings could safeguard against any ‘forgotten’ stents. No cases of stent retention were reported in patients using extraction strings, perhaps supporting the earlier hypothesis. However, it would be advisable to ensure there is some form of follow-up for patients removing their stents at home, such as a phone call from the urology department on the day of planned removal, to reduce the risk of stent retention.

No significant differences in rates of infection or proximal stent migration were reported in any of the four studies, suggesting that perceived risks by clinicians of such complications are unsubstantiated [5, 13–15].

### Tolerability, limitations, cost, and future directions

Removal of stent extraction strings is straightforward and well tolerated by patients. Approximately, 97 % of patients with extraction strings are able to remove them at home without assistance from a healthcare professional [5, 13]. As stated above, pain during removal is comparable with removal in the operating room, with options of anaesthesia and sedation [15]. However, this finding may be subject to selection bias as those opting to have extraction strings may be more confident about the removal process than those who have selected other removal options. Whether increased patient education would result in more patients opting for an extraction string is unclear. However, it is clear that a significant proportion of patients has strong preferences regarding their choice of stent removal method, which should be considered when counselling patients over stent removal options.

An advantage of stent extraction strings is that they reduce healthcare costs, and when used to remove stents at home, it reduces costs associated with patient travel and time taken off work [13]. Barnes et al. estimated avoiding the need for second hospital visit and cystoscopy for stent removal resulted in savings of ~£97,000 in their study population. Bockholt et al. report an estimated $1300/patient cost associated with cystoscopic stent removal, which would be avoided by patients performing home stent extraction using strings [5]. Based on an average 177 mile round trip made by patients for cystoscopic stent removal, Barnes et al. estimated a $68–185 saving per patient on travel costs if patients removed their own stents at home [13]. Such savings may have less impact in smaller countries where distances traveled by patients to their healthcare provider are far less.

Our study was limited to English language and did not include grey literature, potentially leading to a degree of publication bias. Of the studies reviewed, several limitations existed in addition to those stated above. The Barnes et al. study was underpowered due to low patient response rate. It was calculated that a sample size of 76 patients (38 per arm) was required to achieve 80 % power. Unfortunately, this reduces the validity of the only prospective randomized control trial on the use of stent extraction strings. Reasons for under-recruitment suggested to be related to strong preferences over stent removal methods held by patients. Furthermore, it was reported these concerns were higher in men, which may have led to a larger proportion of women to men in the study, which is uncharacteristic of the stone population [13]. Limitations to the Bockholt et al. study include the retrospective study design and lack of validated outcomes measure. Also, the majority of stents with strings were put in by one surgeon, which could result in selection bias [5]. The Loh-Doyle et al. study is limited by potential response bias as survey respondents may not be representative of the stone population. Also, selection bias may have occurred with regard to reported pain outcomes as those with higher anxiety may opt for doctor-stent removal using strings, although this was associated with higher pain scores on removal in their series. Loh-Doyle et al. have compared this to a study on patients undergoing prostate biopsy, in which patients with greater pre-procedure anxiety experienced greater pain during the procedure to increased adrenergic response resulting in hyperalgesia and hypersensitisation of pain receptors [15]. Due to the absence of a control group for comparison, it is difficult to determine the effect of certain findings in the York et al. study, such as pain and retained stones, as it is unclear how much of this was due to the presence of a stent itself rather than extraction strings [16]. As with all systematic reviews, the data were limited to the quality of original studies. In the absence of high-quality studies, with an increasing use of stent with strings, it is perhaps time for powered multicentre studies on this area.

It seems that although in an ideal world uncomplicated URS does not need an indwelling stent, but most urologists prefer to use it possibly as a safety net or as a habitual user. Either way, the ease of stent removal needs to be balanced with the rates of stent dislodgement. It seems prudent that in cases where there is no obvious indication for stent usage, a stent on a string would be a reasonable option. Whereas, in cases with an obvious need for a ureteric stent, such as ureteric injury or perforation or where a relook URS is being considered, a stent without the extraction string would be preferable, so that the patient does not come to any possible harm if the stent was dislodged prematurely. To explore patient and surgeon preferences, future studies also need to address this variation, which might be a reflection of cultural and social acceptance of a ‘string hanging on the outside’.

## Conclusions

Although not universally used following every ureteroscopic procedure, indwelling ureteric stents are commonly inserted at the end of procedures with a view to reducing post-operative morbidity. Their use is often guided by surgeon preference and remains an area of controversy, particularly in groups of patients where there is not a clear-cut indication for routine stent insertion. Until the fields of engineering or pharmacology can provide a safe solution to stent related symptoms, it seems that stent extraction strings can reliably reduce the stent dwell time for patients, thus reducing the duration of morbidity and physical and financial burden to patients. However, this must be balanced against a risk of stent dislodgement and, hence, may not be a good option in all patients.
